# Analysis of Powassan Virus Genome Sequences from Human Cases Reveals Substantial Genetic Diversity with Implications for Molecular Assay Development

**DOI:** 10.3390/v16111653

**Published:** 2024-10-23

**Authors:** Erik H. Klontz, Navid Chowdhury, Nolan Holbrook, Isaac H. Solomon, Sam R. Telford, Matthew T. Aliota, Chantal B. F. Vogels, Nathan D. Grubaugh, Jeffrey Helgager, Holly R. Hughes, Jason Velez, Anne Piantadosi, Charles Y. Chiu, Jacob Lemieux, John A. Branda

**Affiliations:** 1 Department of Pathology, Massachusetts General Hospital and Harvard Medical School, Boston, MA 02114, USA; eklontz@mgh.harvard.edu (E.H.K.); navid_chowdhury@dfci.harvard.edu (N.C.); nolan.holbrook@hmsom.edu (N.H.); 2 Department of Pathology, Brigham and Women’s Hospital and Harvard Medical School, Boston, MA 02115, USA; ihsolomon@bwh.harvard.edu; 3 Department of Infectious Disease and Global Health, Tufts University, North Grafton, MA 02155, USA; sam.telford@tufts.edu; 4 Department of Veterinary and Biomedical Sciences, University of Minnesota, Twin Cities, St. Paul, MN 55455, USA; mtaliota@umn.edu; 5 Department of Epidemiology of Microbial Diseases, Yale School of Public Health, New Haven, CT 06510, USA; 6 Department of Pathology and Laboratory Medicine, School of Medicine and Public Health, University of Wisconsin, Madison, WI 53726, USA; jhelgager@wisc.edu; 7 Centers for Disease Control and Prevention, Fort Collins, CO 80521, USAjdv4@cdc.gov (J.V.); 8 Department of Pathology and Laboratory Medicine, School of Medicine, Emory University, Atlanta, GA 30322, USA; 9 Department of Laboratory Medicine, University of California San Francisco, San Francisco, CA 94115, USA; 10 Department of Medicine, Massachusetts General Hospital and Harvard Medical School, Boston, MA 02114, USA

**Keywords:** Powassan, deer tick virus, lineage I, PCR

## Abstract

Powassan virus (POWV) is an emerging tick-borne virus that causes severe meningoencephalitis in the United States, Canada, and Russia. Serology is generally the preferred diagnostic modality, but PCR on cerebrospinal fluid, blood, or urine has an important role, particularly in immunocompromised patients who are unable to mount a serologic response. Although the perceived poor sensitivity of PCR in the general population may be due to the biology of infection and health-seeking behavior (with short viremic periods that end before hospital presentation), limitations in assay design may also contribute. Genome sequences from clinical POWV cases are extremely scarce; PCR assay design has been informed by those available, but the numbers are limited. Larger numbers of genome sequences from tick-derived POWV are available, but it is not known if POWV genomes from human infections broadly mirror genomes from tick hosts, or if human infections are caused by a subset of more virulent strains. We obtained viral genomic data from 10 previously unpublished POWV human infections and showed that they broadly mirror the diversity of genome sequences seen in ticks, including all three major clades (lineage I, lineage II Northeast, and lineage II Midwest). These newly published clinical POWV genome sequences include the first confirmed lineage I infection in the United States, highlighting the relevance of all clades in human disease. An in silico analysis of published POWV PCR assays shows that many assays were optimized against a single clade and have mismatches that may affect their sensitivity when applied across clades. This analysis serves as a launching point for improved PCR design for clinical diagnostics and environmental surveillance.

## 1. Introduction

Powassan virus (POWV) is an emerging tick-borne flavivirus that is capable of causing severe meningoencephalitis, leading to death in approximately 10% of diagnosed individuals, and long-term neurological sequela in one third of survivors [1]. POWV has long been considered a rare cause of encephalitis, with approximately one case reported per year from 1958 to 2005 [2]. However, the last several years have seen a proliferation of cases, with 211 cases reported in the United States from 2016 to 2022 [3]. The increase in reported cases could be due to increases in infections and/or improved surveillance, but the true incidence is likely still higher, owing primarily to the challenges in making a diagnosis. In the United States, the preferred method in immunocompetent patients is IgM serology with confirmatory plaque reduction neutralization testing. However, this testing is only available in the United States through the CDC and select state/commercial reference laboratories [4]. Real-time reverse transcription-PCR (RT-PCR) from cerebrospinal fluid, blood, or urine is similarly available through select laboratories but is thought to be less sensitive than serology, except in immunocompromised hosts [5,6]. The perceived insensitivity of PCR is due, in part, to the short viremic period in related arboviruses [6]. However, it is not known if the insensitivity of PCR may be influenced by the use of assays that are not optimized to detect the full diversity of POWV genomes, given that many assays were designed before large numbers of POWV genome sequences became available. Recently, diagnoses have also been made through CSF metagenomic next generation sequencing (mNGS), highlighting the usefulness of molecular tests in some patients [7,8,9,10].

Powassan virus has two distinct phylogenetic lineages that are serologically indistinguishable but are spread by different vectors. Lineage I (the prototypical lineage) is spread primarily by *Ixodes cookei* and *Ixodes marxi* ticks; lineage II, also named Deer Tick Virus (DTV) is spread by *Ixodes scapularis* ticks, and can be separated into northeastern and midwestern clades [1]. Both lineages are maintained by distinct enzootic cycles involving small mammals, with humans incidentally infected as dead-end hosts [1]. Hundreds of POWV genome sequences have been determined from ticks, and large phylogenetic studies from ticks have been recently published [11,12]. However, POWV genome sequences from humans are extremely scarce [8,13,14,15,16,17], and it is unknown if human infections are caused by select highly virulent strains, or if they mirror the diversity of strains seen in ticks.

Understanding the genomic diversity of human POWV infections has important implications on the design of sensitive RT-PCR assays. Until now, published POWV genome sequences from human infections in the United States have been exclusively lineage II, with at least nine confirmed cases described [8,13,16,18,19,20,21] and complete or nearly complete genome sequences published for four [8,13]. Lineage I genomes have been detected from humans in Canada and Russia, with sequences published for five [14,15]. Here, we report genome sequences from 10 new human POWV infections, including the first published human lineage I infection in the United States, and demonstrate that they represent the diversity of genomes seen in ticks. An in silico analysis of published RT-PCR assays suggests that many assays were designed against a single lineage or sub-lineage and have mismatches that may limit their sensitivity if applied across sub-lineages.

## 2. Methods

Sequencing and genome assembly. Sequencing and genome assembly were performed at multiple institutions according to researchers’ local preferences. Seven samples (OR130288-OR130294) were sequenced directly from patient CSF using a previously described clinically validated pipeline [22]. Raw mNGS data were aligned to POWV reference genomes (MZ576219.1 for lineage II and MF374486.1 for lineage I) using viral-ngs 2.1.28 hosted on the Terra platform (app.terra.bio). Samples OR130295 and OR130296 were sequenced from patient formalin-fixed paraffin-embedded brain tissue [13,23]: RNA was extracted using the E.Z.N.A FFPE Kit (Omega, Norcross, GA, USA), DNase treated (ArcticZymes, Tromsø, Norway), and converted to cDNA using the SuperScript IV First-Strand Synthesis System (Thermo Fisher Scientific/Invitrogen, Waltham, MA, USA) and New England Biolabs (Ipswich, MA, USA) reagents for second-strand synthesis. Sequencing libraries were fragmented and indexed using the Nextera XT DNA Library Prep kit (Illumina, San Diego, CA, USA) with dual indexes and 16 cycles of PCR. Libraries were quantified using the KAPA Universal Complete Kit (Roche Diagnostics, Indianapolis, IN, USA), pooled to equimolar concentration, and sequenced on an Illumina instrument with paired-end 150–base pair reads. Sample OL695841 was sequenced from BHK-1 cells after one passage from CSF, as previously described [12].

Phylogenetic tree construction. GenBank was queried on 5/8/23 for “Powassan” and all POWV genome sequences between the lengths 8000 and 12,000 nucleotides were downloaded and limited to one genome per patient where duplicates existed. In total, 407 genome sequences were aligned using MAFFT FFT-NS-2 [24]. A maximum likelihood tree was generated with IQ-tree [25] and bootstrap node support calculated using UFBoot2 [26]. For each clinical POWV genome sequence, the percent identity to tick genomes was determined by NCBI blast.

In silico PCR analysis. A representative subset of the genome sequences queried on 5/8/23 was selected for PCR analysis. Up to five complete genomes from each state (Minnesota, Wisconsin, Connecticut, Maine, Massachusetts, New York, New Hampshire, Rhode Island, New Jersey, West Virginia) and country (Canada, Russia) were selected and manually verified for completeness and quality to form a final set of 47 genomes (Supplemental Table S1). Complete clinical genome sequences were prioritized when available. For PCR review, a PubMed search for “Powassan PCR” was conducted on 5/8/23, and papers that described unique primers/probes to detect POWV using RT-PCR were included in this study. Primers/probes were mapped to each genome using Geneious, allowing for up to four mismatch tolerance. Degenerate primers were individually mapped as each of their components, and the result with the fewest mismatches was recorded. When primers bound in multiple locations, the location with the fewest mismatches was used.

Mutation analysis. The same set of 407 genome sequences used for phylogenetic tree construction was assessed for nucleotide and amino acid mutations. Nucleotide sequences were translated using Geneious and analyzed using R. The difference in frequency for each nucleotide and amino acid between samples from ticks and humans was determined and a Fisher’s exact test was performed, with a value considered nominally significant if the two-tailed *p*-value was less than 0.05. When accounting for multiple comparisons, an adjusted significance threshold was determined by dividing 0.05 by the number of comparisons. Analysis was stratified by lineage to account for the relative under-sampling of lineage I tick genomes compared to lineage II tick genomes.

## 3. Results

**Figure 1:**
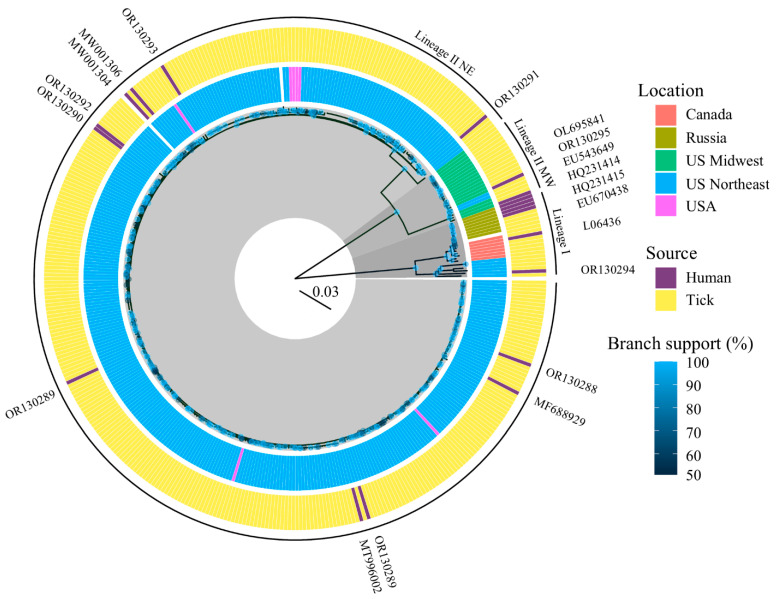
Phylogenetic reconstruction of Powassan virus genome sequences from ticks and humans, colored by location and source. Where node support is >50% by bootstrap approximation, nodes are annotated with blue colored circles shaded by degree of support. Outer rings provide information on the source (tick versus human) and specimen location.

Phylogeny of POWV from humans versus ticks. Powassan virus genomic data were obtained by metagenomic next-generation sequencing of CSF and/or brain tissue from 10 human cases of POWV, producing four partial genomes and six complete genomes. To assess if POWV genomes from human infections broadly sample genomes obtained from ticks, or if they represent a narrow subset of closely related strains, POWV genome sequences from both sources were compared. In total, 19 POWV genome sequences from humans, including 10 newly published, were compared to 387 previously published POWV genomes from ticks. A maximum-likelihood tree revealed that POWV genomes from humans broadly sampled those from ticks (Figure 1). POWV genome sequences from humans clustered within the same three clades that have been previously described in ticks (lineage I, lineage II Northeast, and lineage II Midwest). Among 10 newly published genome sequences, nine belonged to lineage II, with seven falling within to the Northeastern clade, and two within the Midwestern clade, matching the regions in which the patients resided. One new genome belonged to lineage I, coming from a fatal infection in Maine in 2022. The complete genome sequence showed greatest similarity (96.41% nucleotide identity and 98.95% protein identity) to a POWV genome from ticks on *Marmota* spp. in New York in 1964 and showed comparable similarity to other lineage I genome sequences determined more recently from *I. cookei* (95.93% nucleotide identity) and *I. scapularis* ticks (96.12% nucleotide identity) in the US. Excluding gaps, lineage II genome sequences were >99% identical at the nucleotide and protein level to the nearest genome sequences from ticks (Table 1). This is consistent with the observation that most POWV strains are >99% identical at the nucleotide level to other members within their clade (Supplemental Table S2). However, substantial diversity is seen across lineages, with lineage I and lineage II genome sequences sharing as little as 85% nucleotide identity (Table 2).

**Figure 2:**
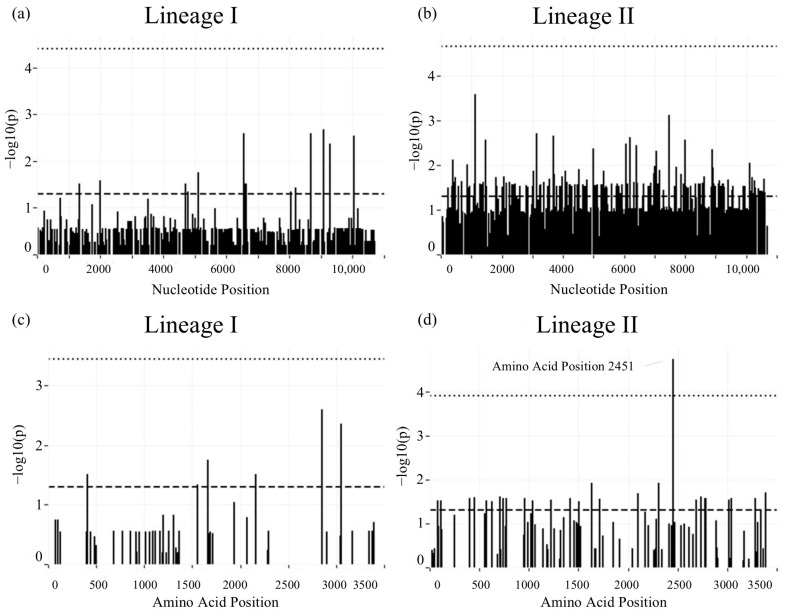
Comparison of POWV genome sequences from humans and ticks by nucleotide and amino acid position by Fisher’s exact test. (**a**) Comparison of lineage I genomes by nucleotide position. (**b**) Comparison of lineage II genomes by nucleotide position. (**c**) Comparison of lineage I genomes by amino acid position. (**d**) Comparison of lineage II genomes by amino acid position. Horizontal dashed line represents threshold for nominal significance (*p* < 0.05); horizontal dotted line represents threshold for significance after correction for multiple comparisons.

*Analysis of mutations*. Substitutions at the amino acid level have led to the emergence and spread of related flaviviruses such as Zika virus and West Nile virus [28,29,30,31]. To assess for mutations that may have led to increased spread or virulence of POWV in humans, the frequency of each nucleotide and amino acid at each position in the genome was compared between genomes from ticks and humans. At the nucleotide level, there were no significant differences at any position between genomes from ticks and humans in lineage I (Figure 2a) or lineage II (Figure 2b) after correcting for multiple comparisons. At the amino acid level, there were no significant differences in lineage I (Figure 2c) but one significant difference (*p* = 1.82 × 10^−5^) at position 2451 in lineage II genomes (Figure 2d). This position, which corresponds to position 191 in NS4b, is an alanine in all genomes from ticks, but a valine (n = 2) or threonine (n = 1) in a subset of genomes from humans.

Analysis of PCR assays. With more POWV genome sequences available, we sought to assess the theoretical suitability of various published PCR assays for the detection of POWV in humans. We performed an in silico analysis of published POWV RT-PCR assays, mapping unique sets of primers/probes to a representative set of genome sequences that samples the known diversity of POWV genomes. The results show that most PCR assays are optimized against a single clade (Table 3). With one-mismatch tolerance in each component (primers and probe), no assay mapped to all genome sequences in the set. The assay described by El Khoury et. al., performed at the New York State lab, had the fewest mismatches of any probe-based assay, mapping with ≤1 mismatch to 98% of analyzed genome sequences (see Supplemental Table S3 for a more detailed summary; primers/probes for the assay performed at Mayo were not available for analysis but detect lineage I and lineage II). Assays performed by the CDC arbovirus branch showed close matching for lineage I and lineage II Midwestern genomes, but relatively less matching for lineage II Northeastern genomes. Two assays targeting the 3′ UTR were negatively influenced in the in silico analysis by the inclusion of complete genomes that terminate before the target region.

## 4. Discussion

Our analysis revealed that POWV genome sequences derived from human infections are highly similar to those derived from ticks, representing the broad diversity of lineage I and lineage II clades in ticks. The similarity between POWV genome sequences from humans and ticks highlights the relevance of environmental surveillance, as RT-PCR assay design for the diagnosis of human infections can be informed by genomic diversity of POWV in ticks. The high similarity between genome sequences from humans and ticks is consistent with the understanding that humans are incidental hosts and that there is minimal viral evolution occurring within humans. The substitution A191V/T in NS4b in some human lineage II genomes is of uncertain significance and requires further investigation. NS4b is a complex and multifunctional protein involved in viral replication and host immune modulation. Homology with related viruses suggests that this position is at the start of the α8 helix, which is likely a transmembrane helix but may undergo conformational changes within the viral lifecycle [39].

Although most human infections belonged to lineage II, our study reports the first lineage I genome sequence derived from a human infection in the United States (note, subsequent to our analysis, a lineage I genome reportedly from a human dating to 1975 was deposited to GenBank under accession OP265689 but has not been published at the time of writing). Although *I. cookei* (groundhog ticks) and *I. marxi* (squirrel ticks) are the primary vectors for lineage I POWV, this lineage has recently been found in *Dermacentor variabilis* (American dog ticks) and *I. scapularis* (deer ticks) [12,40]. The genome sequences from the clinical lineage I infection in this study showed comparable similarity to POWV sequences from *I. cookei* and *I. scapularis*, implicating either of these ticks as the potential source for the patient’s infection. Although *I. cookei* generally does not bite humans as aggressively as *I. scapularis*, in 1989–1990, it was found to account for 34% of human tick bites in Maine [41], the same state in which this patient resided. Although *I. scapularis* has since become more widely distributed, our study highlights the continued importance of POWV lineage I and the need to better understand the relevance of multiple different tick species in contributing to human infections. Compared to lineage II infections that are >99% identical to genomes from ticks, the lineage I infection showed only 96% nucleotide identity to its nearest relative from ticks, suggesting that tick lineage I genomes are relatively under-sampled in environmental surveys.

Our in silico analysis of published RT-PCR assays revealed that diverse strategies have been used to detect POWV, but that most assays have numerous mismatches against specific clades, potentially impacting their sensitivity in detecting those clades. Assays used for clinical diagnosis or environmental surveillance should be designed to detect lineage I, lineage II Northeastern, and lineage II Midwestern clades. The assay published by El Khoury et. al. had the fewest mismatches across all clades, and may serve as a good starting point for investigators when selecting an RT-PCR assay from the literature [21]. There are uncertainties in the sensitivity of assays that target the 3′ UTR, particularly in lineage II NE genomes, as the full length of this region is not present in all genome sequences, including many annotated as complete. Although this is likely due to sequencing/bioinformatic artifacts limiting reads at the 3′ end, it is not known if some viruses have truncated genomes, or if the 3′ end of the genome degrades before sequencing. Our analysis used ≤1 mismatch as the threshold for an ideal match, but assays may tolerate more or fewer mismatches depending on the nature and location of the mismatch. As a noteworthy example, the lineage I infection published here was detected on a clinical basis by the CDC using an envelope-targeted assay despite three mismatches in the forward primer. Although an in silico analysis is a starting point, it is not a replacement for experimental validation, and more studies comparing available RT-PCR assays are required. There is a need for sensitive and specific POWV RT-PCR assays as unbiased mNGS is currently too costly and slow for widespread routine use.

In conclusion, our data show that POWV genomes from ticks are largely identical to those from humans. PCR assays may be designed using genome sequences from ticks, underscoring the importance of environmental surveillance, particularly if lineage I continues to be detected in *I. scapularis*. There is substantial diversity between POWV lineages and clades, which may limit the sensitivity of some existing assays when applied to individuals across large geographic areas. As all lineages are relevant to human disease, the most broadly applicable PCR assays should be designed to detect the full diversity of POWV genomes seen in humans and ticks.

## Figures and Tables

**Table 1:** Comparison of POWV genomes from humans and tick. ^1^ Excluding gaps in partial genomes.

Human Infection	Location	Year	Clade	Closest Tick Genome	Nucleotide Identity (%)	Protein Identity (%)	Citation
OR130294	ME	2022	Lineage I	HM440563	96.41	98.95	This study
OR130288	MA	2022	Lineage II NE	OL704271	99.84	99.97	This study
OR130289	MA	2019	Lineage II NE	OL704356	99.79	99.82	This study, [1,10]
OR130290	MA	2020	Lineage II NE	OP265695	100 ^1^	100 ^1^	This study, [10]
OR130291	MA	2021	Lineage II NE	OL704190	99.34	99.56	This study, [10]
OR130292	ME	2022	Lineage II NE	OP265694	100 ^1^	100 ^1^	This study, [10]
OR130293	MA	2022	Lineage II NE	OL704295	99.39	99.80	This study, [10]
OR130296	ME	2016	Lineage II NE	OL704276	99.90 ^1^	99.89 ^1^	This study
OR130295	WI	2018	Lineage II MW	OP823481	99.50 ^1^	99.85 ^1^	This study
OL695841	MN	2020	Lineage II MW	OP823481	99.35	99.74	This study
HQ231414	RUS	2006	Lineage I	MG652438	99.92	99.94	Unpublished
HQ231415	RUS	2006	Lineage I	MG652438	99.92	99.91	Unpublished
EU543649	RUS	2006	Lineage I	MG652438	99.92	99.85	[14]
EU670438	RUS	1991	Lineage I	MG652438	99.92	99.94	[14]
L06436	CAN	1958	Lineage I	OP823407	99.98	99.91	[15]
MW001304	MA	2016	Lineage II NE	OP823438	99.70	99.88	[1,13,18]
MW001306	MA	2019	Lineage II NE	OP823438	99.75	99.80	[13]
MT996002	MA	2018	Lineage II NE	OL704356	99.87	99.91	[13]
MF688929	NH	2016	Lineage II NE	OP265694	99.89 ^1^	99.82 ^1^	[8,10,27]

**Table 2:** Nucleotide percent identity matrix of clinical POWV genome sequences by lineage.

	Lineage I	Lineage II NE	Lineage II MW
Lineage I	94%	85%	85%
Lineage II NE	85%	99%	94%
Lineage II MW	85%	94%	99%

**Table 3:** Published RT-PCR assays and their frequency of mismatches against lineage I, lineage II Midwest and lineage II Northeast genome sequences.

Primer Set	≤1 Mismatch in All Components	Target	Ref.
Forward-1: ACCATAACAAACATGAAAGTCCAACT Forward-2: CCATCACAAACATGAAAGTCCAACT Reverse-1: TGAGTCTGCTGGTCCGATGAC Reverse-2: CTGTGAGTCAGCTGGTCCTATGAC Probe: 6FAM-CCTTCCATCATGCGGAT-MGB	Lineage I: 92% Lineage II Midwest: 100% Lineage II Northeast: 100% All lineages: 98%	NS5	[21] Assay performed by NYSDOH
Forward: GCATG+A3:G12GTCGGATGAACAGAA Reverse: GAGCGCTCTTCATCCACCA Probe: N/A	Lineage I: 83% Lineage II Midwest: 100% Lineage II Northeast: 100% All lineages: 96%	NS5	[8]
POW-1: TGGATGACAACAGAAGACATGC POW-2: GCTCTCTAGCTTGAGCTCCCA Probe: N/A	Lineage I: 100% Lineage II Midwest: 100% Lineage II Northeast: 88% All lineages: 94%	E	[32]
Forward: CACCAGGAGTTAGGCCATTT Reverse: AGATTGCCAATCTTCTTCCT Reverse: AGATTGCCAATTGTCTTCCC Probe: 6FAM-TCCTCCCGAGTTATGCCCGG-BHQ1	Lineage I: 92% Lineage II Midwest: 100% Lineage II Northeast: 64% All lineages: 78%	3′ UTR	This study. Assay performed by CDC
Forward: GTGATGTGGCAGCGCACC Reverse: CTGCGTCGGGAGCGACCA Probe: Texas Red-CCTACTGCGGCAGCACACACAGTG-BHQ	Lineage I: 0% Lineage II Midwest: 100% Lineage II Northeast: 88% All lineages: 67%	3′ UTR	[33]
Forward: GATCATGAGAGCGGTGAGTGACT Reverse: GGATCTCACCTTTGCTATGAATTCA Probe: 6FAM-TGAGCACCTTCACAGCCGAGCCAG-TAMRA	Lineage I: 0% Lineage II Midwest: 44% Lineage II Northeast: 100% All lineages: 63%	NS5	[34]
Forward: AGAATGGCCATGACAGACACAA Reverse: AGCCAGTCACTCACHGCTCTCAT Probe: ?-GCCCAAGAGCCRCAGCCAGG-?	Lineage I: 100% Lineage II Midwest: 0% Lineage II Northeast: 64% All lineages: 61%	NS5	[35]
Forward: GATCATGAGAGCGGTGAGTGACT Reverse: GGATCTCACCTTTGCTATGAATTCA Probe: 6FAM-TGAGCACCTTCACAGCCGAGCCAG-TAMRA	Lineage I: 0% Lineage II Midwest: 33% Lineage II Northeast: 100% All lineages: 61%	NS5	[36]
Forward: GAAGCTGGGTGAGTTTGGAG Reverse: CCTGAGCAACCAACCAAGAT Probe: N/A	Lineage I: 0% Lineage II Midwest: 0% Lineage II Northeast: 100% All lineages: 54%	NS5	[37]
Forward: GTGCCAAGTTTGAATGCGAGGAAG Reverse: GAACGGGGCCCAGCGAGAGTGAC Probe: N/A	Lineage I: 0% Lineage II Midwest: 0% Lineage II Northeast: 96% All lineages: 52%	NS5	[38]
Forward: CGACCAGCAACGAGCCC Reverse: GCCAAAGAATCCCCAGCAT Probe: 6FAM-CCAAAGGGCTTCGTGCTGTCGC-BHQ1	Lineage I: 100% Lineage II Midwest: 0% Lineage II Northeast: 0% All lineages: 26%	Capsid	This study. Assay performed by CDC
Forward: CAAGCCACACCATCGATAATGA Reverse: CGTTTGCTCACTATATCCAGGTATTC Probe: 6FAM-CTTTTCCTGCCGGTTACTCTCGCCG-BHQ1	Lineage I: 0% Lineage II Midwest: 100% Lineage II Northeast: 0% All lineages: 20%	NS5	This study. Assay performed by CDC
Forward: GCAGTTTACGGTGGCATCC Reverse: CGTCAGCGACACATCTCCAT Probe: 6FAM-AGTGATCCTGCGGCTCGGCG-BHQ1	Lineage I: 75% Lineage II Midwest: 0% Lineage II Northeast: 0% All lineages: 20%	E	This study. Assay performed by CDC
Forward: CATAGCRAAGGTGAGATCCAA Reverse: CTTTCGAGCTCCAYTTRTT Probe: 6FAM-AGCTCTGGGCGCATGGTYGGATGAACA-TAMRA	Lineage I: 0% Lineage II Midwest: 0% Lineage II Northeast: 0% All lineages: 0%	NS5	[34]
ENV-A: GTCGACGACGAGGTGCACGCATCTTGA POW-6: TTGTGTTTCCAGGGCAGCGCCA Probe: N/A	Lineage I: 0% Lineage II Midwest: 0% Lineage II Northeast: 0% All lineages: 0%	NS5	[32]

## Data Availability

Powassan virus genomic data are publicly available at GenBank under accession numbers OR130288-OR130296 and OL695841.

## Supplementary Materials

The following supporting information can be downloaded at: https://www.mdpi.com/article/10.3390/v16111653/s1, Table S1: Genomes used for in silico analysis; Table S2: Pairwise identity of Powassan virus genomes; Table S3: Detailed summary of in silico analysis.

## References

[1] Piantadosi A., Solomon I.H. (2022). Powassan Virus Encephalitis. Infect. Dis. Clin. N. Am..

[2] Kemenesi G., Bányaic K. (2019). Tick-Borne Flaviviruses, with a Focus on Powassan Virus. Clin. Microbiol. Rev..

[3] ArboNET Arboviral Diseases Branch Centers for Disease Control and Prevention Powassan Virus Statistics and Maps. https://www.cdc.gov/powassan/data-maps/historic-data.html.

[4] Clinical Testing and Diagnosis for Powassan Virus Disease. https://www.cdc.gov/powassan/hcp/diagnosis-testing/index.html.

[5] Piantadosi A., Kanjilal S. (2020). Diagnostic Approach for Arboviral Infections in the United States. J. Clin. Microbiol..

[6] Kapadia R.K., Staples J.E., Gill C.M., Fischer M., Khan E., Laven J.J., Panella A., Velez J.O., Hughes H.R., Brault A. (2022). Severe Arboviral Neuroinvasive Disease in Patients on Rituximab Therapy: A Review. Clin. Infect. Dis..

[7] Farrington M., Elenz J., Ginsberg M., Chiu C., Miller S., Pangonis S.F. (2023). Powassan Virus Infection Detected by Metagenomic Next-Generation Sequencing, Ohio, USA. Emerg. Infect. Dis..

[8] Piantadosi A., Kanjilal S., Ganesh V., Khanna A., Hyle E.P., Rosand J., Bold T., Metsky H.C., Lemieux J., Leone M.J. (2018). Rapid Detection of Powassan Virus in a Patient with Encephalitis by Metagenomic Sequencing. Clin. Infect. Dis..

[9] Johnson I.M., Scheckel C., Parikh S.A., Enzler M., Fugate J., Call T.G. (2022). Fatal Powassan Virus Encephalitis in Patients with Chronic Lymphocytic Leukemia. Blood Cancer J..

[10] Klontz E.H., Solomon I.H., Turbett S.E., Lemieux J.E., Branda J.A. (2024). Cerebrospinal Fluid Metagenomics Has Greatest Added Value as a Test for Powassan Virus among Patients in New England with Suspected Central Nervous System Infection. Diagn. Microbiol. Infect. Dis..

[11] McMinn R.J., Langsjoen R.M., Bombin A., Robich R.M., Ojeda E., Normandin E., Goethert H.K., Lubelczyk C.B., Schneider E., Cosenza D. (2023). Phylodynamics of Deer Tick Virus in North America. Virus Evol..

[12] Vogels C.B.F., Brackney D.E., Dupuis A.P., Robich R.M., Fauver J.R., Brito A.F., Williams S.C., Anderson J.F., Lubelczyk C.B., Lange R.E. (2023). Phylogeographic Reconstruction of the Emergence and Spread of Powassan Virus in the Northeastern United States. Proc. Natl. Acad. Sci. USA.

[13] Normandin E., Solomon I.H., Zamirpour S., Lemieux J., Freije C.A., Mukerji S.S., Tomkins-Tinch C., Park D., Sabeti P.C., Piantadosi A. (2020). Powassan Virus Neuropathology and Genomic Diversity in Patients with Fatal Encephalitis. Open Forum Infect. Dis..

[14] Leonova G.N., Kondratov I.G., Ternovoi V.A., Romanova E.V., Protopopova E.V., Chausov E.V., Pavlenko E.V., Ryabchikova E.I., Belikov S.I., Loktev V.B. (2009). Characterization of Powassan Viruses from Far Eastern Russia. Arch. Virol..

[15] Mandl C.W., Holzmann H., Kunz C., Heinz F.X. (1993). Complete Genomic Sequence of Powassan Virus: Evaluation of Genetic Elements in Tick-Borne versus Mosquito-Borne Flaviviruses. Virology.

[16] Tavakoli N.P., Wang H., Dupuis M., Hull R., Ebel G.D., Gilmore E.J., Faust P.L. (2009). Fatal Case of Deer Tick Virus Encephalitis. N. Engl. J. Med..

[17] Feder H.M., Telford S., Goethert H.K., Wormser G.P. (2021). Powassan Virus Encephalitis Following Brief Attachment of Connecticut Deer Ticks. Clin. Infect. Dis..

[18] Solomon I.H., Spera K.M., Ryan S.L., Helgager J., Andrici J., Zaki S.R., Vaitkevicius H., Leon K.E., Wilson M.R., DeRisi J.L. (2018). Fatal Powassan Encephalitis (Deer Tick Virus, Lineage II) in a Patient with Fever and Orchitis Receiving Rituximab. JAMA Neurol..

[19] Cavanaugh C.E., Muscat P.L., Telford S.R., Goethert H., Pendlebury W., Elias S.P., Robich R., Welch M., Lubelczyk C.B., Smith R.P. (2017). Fatal Deer Tick Virus Infection in Maine. Clin. Infect. Dis..

[20] El Khoury M.Y., Camargo J.F., White J.L., Backenson B.P., Dupuis A.P., Escuyer K.L., Kramer L., George K.S., Chatterjee D., Prusinski M. (2013). Potential Role of Deer Tick Virus in Powassan Encephalitis Cases in Lyme Disease-Endemic Areas of New York, USA. Emerg. Infect. Dis..

[21] El Khoury M.Y., Hull R.C., Bryant P.W., Escuyer K.L., St George K., Wong S.J., Nagaraja A., Kramer L., Dupuis A.P., Purohit T. (2013). Diagnosis of Acute Deer Tick Virus Encephalitis. Clin. Infect. Dis..

[22] Miller S., Naccache S.N., Samayoa E., Messacar K., Arevalo S., Federman S., Stryke D., Pham E., Fung B., Bolosky W.J. (2019). Laboratory Validation of a Clinical Metagenomic Sequencing Assay for Pathogen Detection in Cerebrospinal Fluid. Genome Res..

[23] Piantadosi A., Shariatzadeh N., Bombin A., Arkun K., Alexandrescu S., Kleinschmidt-Demasters B.K., Solomon I.H. (2023). Double-Stranded RNA Immunohistochemistry as a Screening Tool for Viral Encephalitis. Am. J. Clin. Pathol..

[24] Katoh K., Standley D.M. (2013). MAFFT Multiple Sequence Alignment Software Version 7: Improvements in Performance and Usability Article Fast Track. Mol. Biol. Evol..

[25] Nguyen L., Schmidt H.A., von Haeseler A., Minh B.Q. (2014). IQ-TREE: A Fast and Effective Stochastic Algorithm for Estimating Maximum-Likelihood Phylogenies. Mol. Biol. Evol..

[26] Thi Hoang D., Chernomor O., von Haeseler A., Quang Minh B., Sy Vinh L., Rosenberg M.S. (2017). UFBoot2: Improving the Ultrafast Bootstrap Approximation. Mol. Biol. Evol..

[27] Piantadosi A., Mukerji S.S., Ye S., Leone M.J., Freimark L.M., Park D., Adams G., Lemieux J., Kanjilal S., Solomon I.H. (2021). Enhanced Virus Detection and Metagenomic Sequencing in Patients with Meningitis and Encephalitis. mBio.

[28] Shan C., Xia H., Haller S.L., Azar S.R., Liu Y., Liu J., Muruato A.E., Chen R., Rossi S.L., Wakamiya M. (2020). A Zika Virus Envelope Mutation Preceding the 2015 Epidemic Enhances Virulence and Fitness for Transmission. Proc. Natl. Acad. Sci. USA.

[29] Brault A.C., Huang C.Y.-H., Langevin S.A., Kinney R.M., Bowen R.A., Ramey W.N., Panella N.A., Holmes E.C., Powers A.M., Miller B.R. (2000). A Single Positively Selected West Nile Viral Mutation Confers Increased Virogenesis in American Crows Aaron. Nat. Genet..

[30] Xia H., Luo H., Shan C., Muruato A.E., Nunes B.T.D., Medeiros D.B.A., Zou J., Xie X., Giraldo M.I., Vasconcelos P.F.C. (2012). An Evolutionary NS1 Mutation Enhances Zika Virus Evasion of Host Interferon Induction. Nat. Commun..

[31] Moudy R.M., Meola M.A., Morin L.L.L., Ebel G.D., Kramer L.D. (2007). A Newly Emergent Genotype of West Nile Virus Is Transmitted Earlier and More Efficiently by Culex Mosquitoes. Am. J. Trop. Med. Hyg..

[32] Telford S., Armstrong P., Paula K., Foppa I., Olmeda Garvia S., Wilson M., Spielman A. (1997). A New Tick-Borne Encephalitis-like Virus Infecting New England Deer Ticks, Ixodes Dammini. Emerg. Infect. Dis..

[33] Tokarz R., Tagliafierro T., Cucura D.M., Rochlin I., Sameroff S., Lipkin W.I. (2017). Detection of Anaplasma and Powassan Virus in Ticks by a Multiplex Real-Time Reverse Transcription-PCR Assay. mSphere.

[34] Dupuis A.P., Peters R.J., Prusinski M.A., Falco R.C., Ostfeld R.S., Kramer L.D. (2013). Isolation of Deer Tick Virus (Powassan Virus, Lineage II) from Ixodes Scapularis and Detection of Antibody in Vertebrate Hosts Sampled in the Hudson Valley, New York State. Parasites Vectors.

[35] Aliota M.T., Dupuis A.P., Wilczek M.P., Peters R.J., Ostfeld R.S., Kramer L.D. (2014). The Prevalence of Zoonotic Tick-Borne Pathogens in Ixodes Scapularis Collected in the Hudson Valley, New York State. Vector-Borne Zoonotic Dis..

[36] Brackney D.E., Nofchissey R.A., Fitzpatrick K.A., Brown I.K., Ebel G.D. (2008). Short Report: Stable Prevalence of Powassan Virus in Ixodes Scapularis in a Northern Wisconsin Focus. Am. J. Trop. Med. Hyg..

[37] Schwartz S., Calvente E., Rollinson E., Koon D.S.K., Chinnici N. (2022). Tick-Borne Pathogens in Questing Blacklegged Ticks (Acari: Ixodidae) from Pike County, Pennsylvania. J. Med. Entomol..

[38] Yuan Q., Llanos-Soto S.G., Gangloff-Kaufmann J.L., Lampman J.M., Frye M.J., Benedict M.C., Tallmadge R.L., Mitchell P.K., Anderson R.R., Cronk B.D. (2020). Active Surveillance of Pathogens from Ticks Collected in New York State Suburban Parks and Schoolyards. Zoonoses Public Health.

[39] Wang Y., Xie X., Shi P.Y. (2022). Flavivirus NS4B Protein: Structure, Function, and Antiviral Discovery. Antivir. Res..

[40] Lange R.E., Dupuis II A.P., Prusinski M.A., Maffei J.G., Koetzner C.A., Ngo K., Backenson B., Oliver J., Vogels C.B.F., Grubaugh N.D. (2023). Identification and Characterization of Novel Lineage 1 Powassan Virus Strains in New York State. Emerg. Microbes Infect..

[41] Smith R.P., Lacombe E.H., Rand P.W., Dearborn R. (1992). Diversity of Tick Species Biting Humans in an Emerging Area for Lyme Disease. Am. J. Public Health.

